# Depression among type 2 diabetic insulin-dependent older adults

**DOI:** 10.1192/j.eurpsy.2022.1689

**Published:** 2022-09-01

**Authors:** F. Zaouali, N. Lassoued, F. Boubaker, W. Alaya, M.H. Sfar

**Affiliations:** University hospital of Mahdia, Endocrinology Department, Mahdia, Tunisia

**Keywords:** Insulin, Aged, Depression, Diabetes Mellitus, Type 2

## Abstract

**Introduction:**

Although depression is one of the most common diseases among older people, it is still underdiagnosed due to frequent misleading symptoms.

**Objectives:**

The aims of our study were to assess depression in type 2 diabetic insulin-dependent older adults and to identify factors associated with depression among this population.

**Methods:**

A cross-sectional study on 100 type 2 diabetic insulin-dependent elderly recruited from the outpatient endocrinology consultation during June and July 2021. We applied the geriatric assessment scores: the Geriatric Depression Scale 15-item, the KATS score, the Lawton scale. the five-word test, the Mini Nutritional Assessment and the Timed Up and Go test.

**Results:**

The mean age of the population was 70.8±5.8 years with sex ratio of 0.85. Depression was noted among 57% of the patients who were distributed as follow: around one fifth (21%) had mild depression while 36% had moderate to severe depression. Around one quarter of the patients (24%) were dependent in the basic activities of daily living. Depression was significantly associated with dependency (β = 5.27; 95% CI, 1.01 to 27.35), ophthalmologic diseases (β = 8.81; 95% CI, 2.18 to 35.63), high frequency of nocturia (β = 3.71; 95% CI, 1.24 to 11.05) and high frequency of bleeding at insulin injection site (β = 4.21; 95% CI, 1.49 to 11.84).

**Conclusions:**

Our findings suggest that the prevalence of depression is high among type 2 diabetic insulin-dependent older adults. Early assessment of depression’s risk factors is a major pillar of the comprehensive care of our seniors.

**Disclosure:**

No significant relationships.

